# Ancient schwannoma of the vagus nerve, resection with continuous monitoring of the inferior laryngeal nerve

**DOI:** 10.1016/S1808-8694(15)31108-3

**Published:** 2015-10-19

**Authors:** Claudio Gilberto Yuji Nakano, Luiz Claudio Bosco Massarollo, Erivelto Martinho Volpi, José Geraldo Barbosa Junior, Vitor Arias, Rubens Yassuzo Ykko Ueda

**Affiliations:** 1 Medical student, FCMSCSP; 2 Chief of the Head & Neck Surgery Unit, Sao Cristovao Hospital and Guarulhos Oncology Institute; 3 Assistant physician - Head & Neck Surgery Unit, HCFMUSP; 4 Assistant physician - Head & Neck Surgery Unit, Sao Cristovao Hospital; 5 Pathologist, Adolfo Lutz Institute and FMUSP; 6 Surgeon, Head & Neck Surgery Unit, Sao Cristovao Hospital. Sao Cristovao Hospital and Guarulhos Oncology Institute

**Keywords:** intra-operative eletrophysiologic monitoring, recurrent laryngeal nerve, vagus nerve, neurinnoma, ancient schwannoma

## INTRODUCTION

Schwannomas (neurinomas, neurilemmomas) are benign, single, slow-growing encapsulated tumors that originate in the sheath of cranial or spinal nerves,^1^ and that rarely undergo malignant transformation.

Descriptions have shown that about 25% of cases occur in the head and neck;^2^ there are only 95 references of vagus nerve involvement.^3^ These tumors appear mostly between the third and fifth decades of life; there is no sex predominance.^4^ The clinical picture usually consists of a relatively pain-free bulge in the neck; the differential diagnosis should be made with other parapharyngeal tumors or neoplasms in the jugular foramen.^3^

The senile schwannoma (SS) is a rare variant that was first described by Ackrman and Taylor in 1951;^2^ its features are: wide areas of hyalinized matrix, hypercellularity with nuclear polymorphism and cell hyperchromatism. A microscopic description of SS in serial and histological sections reveals two cell types: the Antoni type A or fasciculated type (elongated cells, arranged in intertwining bundles in various directions or in a spiral layout), and the Antoni type B or reticular type (polymorphic cells that define small vacuoles, giving the tumor a honeycomb aspect). Antoni type B cells predominate in SS. Absence of mitosis is the main feature that differentiates a SS from a malignant schwannoma. Twelve cases of head and neck SSs have been described so far, of which one involved the vagus nerve.^5^

Surgery is the treatment of choice; there is a high rate of vagus nerve injury during this procedure.^3^ There are descriptions of resections of vagus nerve schwannomas associated with neurostimulation^3^^,^^6^ and observation of esophageal6 contractions or endoscopic visualization of the larynx.^3^ The current article is the first case report of resection of a vagus nerve schwannoma under continuous electrophysiological monitoring of the recurrent laryngeal nerve.

## CASE REPORT

**Figure 1:**
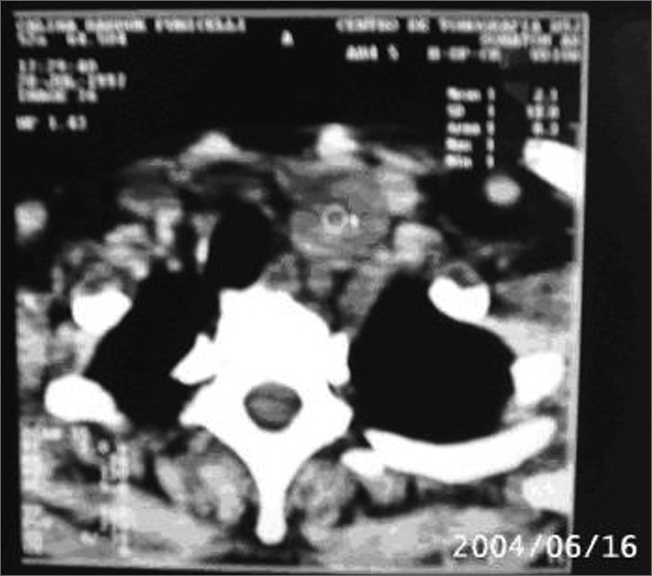
Computed tomography of the cervico-thoracic area (coronal slice) showing a tumor next to the trachea and the lower pole of the left thyroid lobe.

A female, 59-year-old patient reported a 10-year history of multinodular goiter and a palpable nodule in the left supraclavicular fossa. She complained of coughing upon flexing the neck and upon percussion of the supraclavicular nodule, which was gradually worsening. Computed tomography revealed a nodule in the cervical-thoracic transition point, juxtaposed to the trachea and the left lower pole of the thyroid (Figure 1). Fine needle aspiration was done for cytology, which suggested a mesenchymal tumor.

The patient was operated on 21 June 2004; the initial procedure was a total thyroidectomy and dissection with preservation of the recurrent laryngeal nerves. At this point an encapsulated tumor was found close to the lower pole of the left thyroid lobe, which extended retrosternally.

The vagus nerve tumor was completely removed under continuous monitoring (NIM-2® System); laryngeal innervation was preserved. The patient was discharged on the first postoperative day with no intercurrences; direct laryngoscopy after the surgical procedure revealed normally functioning vocal folds. Three years after surgery there are no signs of recurrence or changes in phonation.

Histopathology showed areas of vacuolization, increased cellularity, pleomorphism and hyalinization. Immunohistochemistry was strongly reactive for vimentin and the S-100 protein, which confirmed the diagnosis of SS.

## DISCUSSION

Primary tumors of the vagus nerve are uncommon. Schwannomas are infrequent and the SS variant has been described previously only once.^3^

Surgery has a high rate of vocal fold injury and paralysis, particularly in tumors located close to the jugular foramen.^2^

Fujino^6^ (2000) described the intracapsular enucleation technique for vagus nerve tumors, which has become the standard surgical method - together with neurostimulation - for the treatment of these tumors.

Mevio^2^ (2003) reported vagus nerve tumor resection with neurostimulation and endoscopic observation of the ipsilateral vocal fold. The use of electrodes together with endotracheal ventilation tubes for continuous intra-operative monitoring during thyroidectomy has been well described in the literature.^7^ This system makes possible a simplified non-invasive technique that is just as sensitive as laryngeal muscle monitoring.^7^

This is the second report of a vagus nerve SS and the first report of a case in which continuous laryngeal nerve electrophysiological monitoring was used when resecting a primary vagus nerve tumor.

## CONCLUSION

Schwannomas should be included in the differential diagnosis of vagus nerve tumors. Whenever possible, surgical removal of these tumors should include continuous intraoperative electrophysiological monitoring of the laryngeal nerve.
